# An Ideal Dental Laboratory

**Published:** 1904-12-15

**Authors:** Charles H. Worboys


THE
DENTAL REGISTER.

Vol. LVIII. December 15, 1904. No. 12.

### AN IDEAL DENTAL LABORATORY.

BY CHARLES H. WORBOYS, D.D.S.

Read before the Michigan State Dental Association, June 29th, 1904.

An ideal laboratory is probably an impossibility. Our offices are seldom built for our purposes, and we have to be contented with what happens to be arranged for us. For this reason our laboratories are often so far from ideal that we cannot even consider them good.

In my endeavor to describe an ideal laboratory, I shall try to place before you such a laboratory as a dentist should use, who does every sort of work that may be required of him. The many tools and appliances that are used by us for the work that is done in this section of the office will not be taken up in detail, as it would require a paper far too long for this occasion, but some will be considered.

The location of the laboratory should be among the first things decided upon in arranging the office. The proper place for this room is to the right of the operating room, and it should be at least twelve by fifteen feet in size. A room of this size may seem to some to be far too large for convenience, but when it is remembered that one has to spend hours in this room, we can readily see that it is, if anything, too small for good hygienic conditions. The work to be done at the different benches will require different sets of tools and appliances, and can be spread around a good-sized room and still be very convenient.

This room should be well supplied with light which comes in through at least two large windows at the front. I wish especially to call your attention to this point, as I have seen so many laboratories tucked away in a little dark hole, where it is simply impossible for any man to do that fine work which is required of us. No one realizes this more than I from an experience of about twelve years with my laboratory lighted mainly by a skylight, while I gave up two large front windows for my patients to sit at perhaps not more than fifteen minutes while waiting for me, as a reception room.

I made a change, putting my reception room under the skylight and the laboratory to the front. No patient has found fault with the location of the reception room, and I certainly have enjoyed the light and the convenience.

After the location comes the arrangement, which, of course, depends somewhat on the line of work that one usually does. We shall, however, construct such a laboratory as one needs for every sort of prosthetic work. The floor should be made smooth and then covered with linoleum of a single piece, if possible, and of a brownish color as it wears better and is easier cleaned. The door is best as near the operating chair as possible, with a bench at the left of it toward the front window. This bench should be used exclusively for the porcelain work and must be kept clean at all times, and nothing allowed on it that might mix with the porcelain powder. I keep a clean white paper on mine. All benches that are intended to sit at while working should be no higher than thirty inches, so that we can sit in a common hard-seated revolving chair that has a back. High stools should have no place in the laboratory, as we stand on our feet very much of the time while at the chair.

To the right of the door is a most desirable position for the plaster bench, so as to have it as near to the chair as possible. Above this bench may be hung the bins for plaster, and the impression trays kept in a drawer just below. The plaster bench should have a hole near the wall with a hard brass plate around it, a trough leading from the hole to a receptacle below for refuse. On top of this bench a piece of plate glass should be kept to build models on. Next to the plaster bench is where we need the sink, and it must have a very good trap below it or there will be trouble when the waste pipe fills with bits of plaster. Next to the sink should be the bench used for vulcanite work. The entire apparatus may be centralized in one bench and kept separate from other work. By being convenient to the sink and polishing lathe it will be practicable to do this work without soiling the person or laboratory. The plaster bench, sink and vulcanite work bench might occupy one whole side of the room.

At the front where the light is strong is an ideal place for crown and bridge-work and the arrangement of teeth for artificial dentures, as this line of work calls for very nice fitting and there is no better place for it, and the grinding lathe should be here, too, for convenience.

Benches for the different lines of work are now made and very well adapted to the various uses for which they are designed, and I believe that one, fitting up a laboratory, would be able to do better by getting some of them than to try and have them built. One-half the money spent in a laboratory that is spent in an operating room would make a far different looking place of it than the most of us have. As perhaps a third of our work is done in this room, I am surprised at its shiftless arrangement many times seen.

Along the opposite wall there should be a fire-proof bench for soldering, casting and melting metals. This bench is made of gas pipe with a fire-brick top and a back of about eight inches, and should be at least three feet long. Next to this one, toward the back of the room, can be arranged for the mold'ng as it will be close to the casting bench. Gas for soldering with the bellows under the bench, or compressed air for the blast when one has the apparatus. Zinc and lead can be melted over gas with the Bunsen flame. The electric arc is a valuable adjunct to the laboratory, as with it platinum can be melted and the scrap used. Gasoline has now been harnessed so well that one can do anything with it that can be done with illuminating gas. For porcelain work, the results with the gasoline furnace equal those obtained with anything else. The electric furnace is about all that one can ask to fuse porcelain, and can be used for other purposes. There are on the market small heating stoves that I would prefer to a furnace built in the room for melting and refining, and can be placed in the back part of the room and used for all purposes of a stove.

The polishing lathe should be as far from the porcelain bench as possible and yet have light enough to see to do the work well. Finished work is very important. The rolls need not be very close to the front as that work is all gauged. A good swaging block of solid timber, twelve inches in diameter at the base and eight at the top, thirty inches high, or an anvil in a box of sand, with a good heavy hammer handy by, cannot be dispensed with.

At some convenient place should be located a rest couch, about six feet long and two feet wide, well upholstered and made as comfortable as possible. A ten minutes' rest, lying on one's back with the eyes closed and the mind free, will prepare one to meet the next patient, after a hard struggle which has drawn severely on one's strength. The laboratory will make it practicable to get this rest free from intrusion. The laboratory should also have a convenient place for a few choice books and other things which may be useful in spending idle time pleasurably as well as profitably. A well-equipped laboratory may serve an excellent means for cultivating friendly professional relations with our confreres.

A laboratory of good size, equipped with convenient benches and modern appliances, with good ventilation and light, will certainly enable one to do his best for his patients. It will be a pleasure to work in it and the laboratory work will not be despised.

DISCUSSION.

Dr. E. C. Moore: I like the suggestions of the essayist as to size of the laboratory and good light. A corner room makes an ideal laboratory. Then you can get both light and ventilation. I fully agree with the essayist that we spend an undue amount of money in furnishing our reception rooms, which, in a well-conducted practice, serve only as places for short waitings of patients, and for changing wraps. Some easy chairs, a few good books, a place for experimental research in mechanical lines, such as a general work bench with a nice turning lathe for metal work or such tools as can be afforded, will be the means of spending many idle hours entertainingly if not to great advantage. The laboratory can be made a very hospitable place, and it might not be amiss to include an ice-box in the list of necessary appliances.

Dr. J. F. Spring: I would suggest that a light-colored floor covering is much preferrable to a dark-brown one, as it is much easier to find small articles which one is likely to drop. A kitchen cabinet is a very convenient piece of furniture for a laboratory as it gives places for conveniently storing the numerous things which naturally accumulate in a laboratory.

Dr. Isaac Douglas: I do not care for so large a laboratory as the essayist describes. My laboratory is just seven and one-half feet square, and has but one window, which is, however, forty inches wide. My laboratory is just off my operating room to the right. I have benches all around the room, the general work bench is immediately in front of the window. My plaster bench and vulcanizer bench are together and close to the door. I can sit at my work bench and without leaving my chair put my hands on almost everything in the room. My lathe is run bya water motor. I believe I can do my work as thoroughly with this compact arrangement as I could with more latitude.

Dr. Hoff: I can most cordially agree with the idea of a sufficiently commodious laboratory to accommodate all the necessary appliances for doing all kinds of modern work. It is surprising what meager and old worn-out appliances some good dentists are content to work with. I recently visited an office which reminded one of a junk shop more than a dental laboratory. It contained almost everything from a wornr-out bird cage to a static electric machine, except that the dental appliances were few and badly worn out. The old foot lathe was so badly worn that it rattled badly. A battered vulcanizer and a few worn-out files and scrapers were practically all the tools visible. There was dust and dirt everywhere. It was a poor place for sociability and its occupant was not notorious as a sociable person. It is not necessary that our laboratories should be filled with all manner of devices, however useful they may be on occasions, but enough well-selected and up-to-date appliances, necessary to do modern work should be found is every well-regulated laboratory. I like the suggestion of Dr. Moore, that we have a good turning lathe and such tools as we can use along with it. I know a dentist who has a photographing outfit in his laboratory which gives him a lot of pleasure. We should not of course clutter our laboratory up with too many things, but we often have a few minutes' time that we can throw into some outside work to advantage if prepared for it.

Dr. Worboys: I can readily see that practitioners in large cities can not pay the rents necessary for the large laboratories I have described for you. This is to be regretted. I could not do my best work in a laboratory the size of Dr. Douglas'. I need lots of fresh air and I couldn't get it in his seven by seven room, even if I was able to sit in the middle of it and reach all around it; I would feel so cramped that I cou'd not speed my work. I don't think we have any right to hazard our health for the sake of turning out work rapidly. I keep an extra chair always for any dentist who might chance to come along and wish to call on me. I hadn't thought of an ice-box, but will ponder Dr. Moore's suggestion and may add one for sociable purposes.

